# Fe-phyllosilicate redox cycling organisms from a redox transition zone in Hanford 300 Area sediments

**DOI:** 10.3389/fmicb.2013.00388

**Published:** 2013-12-16

**Authors:** Jason Benzine, Evgenya Shelobolina, Mai Yia Xiong, David W. Kennedy, James P. McKinley, Xueju Lin, Eric E. Roden

**Affiliations:** ^1^Department of Geoscience, University of Wisconsin MadisonMadison, WI, USA; ^2^Pacific Northwest National LaboratoryRichland, WA, USA; ^3^School of Biology, Georgia Institute of TechnologyAtlanta, GA, USA

**Keywords:** subsurface, sediment, microbial, phyllosilicate, iron, redox, enrichment, isolation

## Abstract

Microorganisms capable of reducing or oxidizing structural iron (Fe) in Fe-bearing phyllosilicate minerals were enriched and isolated from a subsurface redox transition zone at the Hanford 300 Area site in eastern Washington, USA. Both conventional and in situ “i-chip” enrichment strategies were employed. One Fe(III)-reducing *Geobacter* (*G. bremensis* strain R1, *Deltaproteobacteria*) and six Fe(II) phyllosilicate-oxidizing isolates from the *Alphaproteobacteria* (*Bradyrhizobium japonicum* strains 22, is5, and in8p8), *Betaproteobacteria* (*Cupriavidus necator* strain A5-1, *Dechloromonas agitata* strain is5), and *Actinobacteria* (*Nocardioides* sp. strain in31) were recovered. The *G. bremensis* isolate grew by oxidizing acetate with the oxidized form of NAu-2 smectite as the electron acceptor. The Fe(II)-oxidizers grew by oxidation of chemically reduced smectite as the energy source with nitrate as the electron acceptor. The *Bradyrhizobium* isolates could also carry out aerobic oxidation of biotite. This is the first report of the recovery of a Fe(II)-oxidizing *Nocardioides*, and to date only one other Fe(II)-oxidizing *Bradyrhizobium* is known. The 16S rRNA gene sequences of the isolates were similar to ones found in clone libraries from Hanford 300 sediments and groundwater, suggesting that such organisms may be present and active *in situ*. Whole genome sequencing of the isolates is underway, the results of which will enable comparative genomic analysis of mechanisms of extracellular phyllosilicate Fe redox metabolism, and facilitate development of techniques to detect the presence and expression of genes associated with microbial phyllosilicate Fe redox cycling in sediments.

## Introduction

Subsurface sediments and groundwater at the 300 Area of the Hanford Site in southeastern Washington State are contaminated with large quantities of radioactive waste generated during Cold War Era nuclear weapons production. Process wastewater infiltrated through the ca. 10-m-thick vadose zone below the disposal facilities, leading to a groundwater uranium (U) plume that has persisted for decades (Peterson and Connelly, 2001; Christensen et al., 2004). Other subsurface environments at Hanford are similarly contaminated with large quantities of technetium (Tc)-99 (Zachara et al., 2007). The valence state of U and Tc is a crucial factor determining their mobility in the subsurface. Both U and Tc are typically present as soluble anionic species under oxic conditions, but can be converted to insoluble UO_2_ (uraninite) and TcO_2_ (technetium dioxide) phases through both biological and abiotic processes under anoxic conditions (Borch et al., 2010). Iron (Fe) bearing mineral phases are likely to play a central role in controlling the U and Tc stability, e.g., through (1) oxide- or phyllosilicate-associated Fe(III) serving as an electron acceptor for dissimilatory metal-reducing bacterial (DMRB) that are capable of simultaneous enzymatic Fe(III) and U(VI) or Tc(VI) reduction (e.g., Jeon et al., 2004; Burke et al., 2005); or (2) Fe(II)-bearing phases, potentially biogenic in origin, serving as abiotic reductants for U(VI) or Tc(VI) (e.g., Fredrickson et al., 2004; Jeon et al., 2005; Lee et al., in press).

The upper, unconfined Hanford 300 Area sediments are comprised of relatively unweathered, Pleistocene-age glacial deposits of the Hanford formation. These sediments are dominated by basaltic and granitic fragments with interspersed silt and clay-sized phyllosilicates (chlorites and ferruginous biedellites as well as some smectite) (Zachara et al., 2007). The older, Miocene-Pliocene-age Ringold Formation that underlies the Hanford formation contains more weathered sediments (Lindsey and Gaylord, 1990), which are dominated by dioctahedral smectite with traces of chlorite, kaolinite, illite, quartz, cristabolite, and feldspar (Peretyazhko et al., 2012). Oxic-anoxic transition zones are observed in fine-grained Ringold Formation sediments, which are likely the result of (at least in part) microbially-driven processes. In particular, there is a distinct redox transition near the top of the Ringold formation (Lin et al., 2012a; Peretyazhko et al., 2012), below which a variety of DMRB taxa have been identified using molecular methods (Lin et al., 2012a). Wet-chemical and spectroscopic analyses suggest that the transition from oxic to anoxic layers involves significant, presumably microbially-catalyzed, reduction of Fe(III) in phyllosilicates (Peretyazhko et al., 2012). Fe(II)-bearing phyllosilicates in Ringold sediments are potent reductants for Tc(VI) (Fredrickson et al., 2004; Peretyazhko et al., 2012), and hence the redox transition zone in the upper Ringold represents a potentially important barrier toward vertical Tc migration. Whether or not microbial activity plays a role in the oxidative transformation of Fe(II)-bearing phases (e.g., reduced phyllosilicates) in the vicinity of the redox transition is unknown.

The purpose of this study was to isolate and identify microorganisms associated with Fe-phyllosilicate redox metabolism in Ringold formation clays and Hanford 300 Area groundwater. While the capacity for DMRB to reduce structural Fe(III) in phyllosilicates is well-established, much less is known about the potential for microbially-catalyzed oxidation of Fe(II)-bearing phyllosilicates (Dong et al., 2009). A key goal of the current work thus was to evaluate, using culture-based approaches, whether or not Hanford 300 Area sediment contains organisms that are capable of enzymatic oxidation of structural Fe(II) in clays and other Fe-silicate minerals. We also screened for the presence of Fe(III) phyllosilicate-reducing organisms. Information on the phylogenetic and physiological properties of Fe phyllosilicate redox cycling bacteria would be useful for developing tools to track the presence, abundance, and activity of Fe redox cycling organisms in the environment. In particular, such tools would be valuable for determining the role that such organisms may play in controlling the coupled redox speciation of Fe and metal/radionuclide contaminants such as U and Tc in subsurface sediments at Hanford and other U.S. DOE sites.

## Materials and methods

### Field site and materials

**Figure A1:**
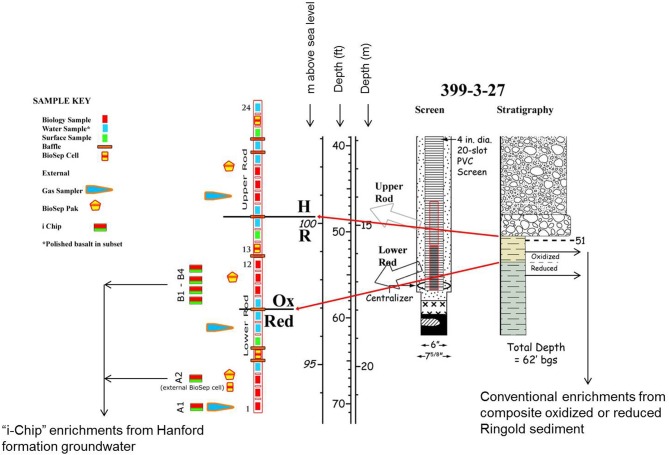
**Diagram illustrating the 300 Area subsurface stratigraphy, sampling strategies, and sources of microbial inocula for this study**.

Sediments from the Ringold formation and groundwater from the Hanford and Ringold formations were sources of inocula for microbial enrichment and isolation. Figure A1 provides an overview of the stratigraphy, well-configuration, and sampling strategies employed for studies of the Hanford 300 Area subsurface. Detailed descriptions of the Hanford 300 Area subsurface environment are available elsewhere (Zachara et al., 2007; Lee et al., 2012; Lin et al., 2012a; Peretyazhko et al., 2012). Two sediments from the Ringold formation were obtained from archived, refrigerated cores sections: (1) “oxidized” material from above the redox transition that was a composite of samples collected from cores C6197 and C6200 (depths interval 17.7–18.0 m); and (2) “reduced” material that was a composite of samples collected from cores C6186, C6190, and C6200 (depths interval 18.6–18.9 m). Groundwater was collected in October 2009 from well 399-3-27, which extends into the redox transition zone in the upper Ringold formation.

### Microbial enrichment strategies

**Figure A2:**
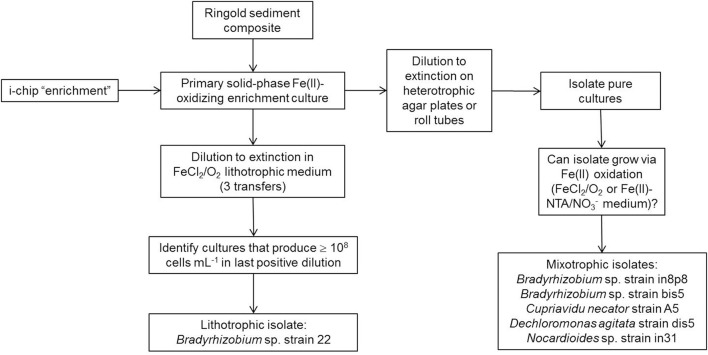
**Strategy for enrichment and isolation of Fe(II)-oxidizing bacteria**.

Two different approaches were employed to recover phyllosilicate-Fe redox cycling organisms: conventional enrichment culture approaches using Ringold sediment composites, and an *in situ* “i-chip” mineral incubation procedure adapted from that described for use with various types of environmental samples (Nichols et al., 2010). Figure A2 provides a schematic view of these procedures as applied to the enrichment and isolation of chemolithoautotrophic and mixotrophic Fe(II)-oxidizing organisms.

#### Conventional enrichments

Fe(III)-reducing enrichment cultures were initiated in anaerobic pressure tubes containing 9 mL of anaerobic bicarbonate-buffered medium containing (g/liter) NaHCO_3_ (2.5), NH_4_Cl (0.25), and NaH_2_PO_4_ · H_2_O (0.06). Medium was dispensed into 25-mL pressure tubes and bubbled with N_2_-CO_2_ (80:20), resulting in a final pH of ca. 6.8. The tubes were capped with butyl rubber stoppers and sterilized by autoclaving. A small quantity of FeCl_2_ (1.3 mM) was added as a reducing agent following sterilization, after which 0.5 g of oxidized Ringold sediment from just above the redox transition zone was added to provide both a microbial inoculum and a source of Fe(III) (present as a mixture of Fe(III) oxides and phyllosilicates) as an electron acceptor. Hydrogen (5 mL in the headspace) and acetate (5 mM) were provided as a combined electron donor and carbon source for the Fe(III)-reducing enrichments.

Fe(II)-oxidizing enrichments were established with reduced Ringold material and either nitrate or oxygen as the electron acceptor in Pipes-buffered medium containing (g/L): Pipes (3.35), NH_4_Cl (0.25), NaH_2_PO_4_.H_2_O (0.06), KCl (0.1). In addition to the reduced Ringold material, the specimen minerals biotite (a primary Fe(II)-bearing silicate mineral) or reduced NAu-2 smectite (a secondary phyllosilicate mineral) were used as a source of Fe(II) in some enrichment cultures. Both biotite and reduced smectite have been shown to be suitable sources of the electron donor for lithotrophic microbial metabolism (Shelobolina et al., 2003, 2012a,b). Biotite is stable toward air oxidation and was therefore utilized as the electron donor for aerobic Fe(II)-oxidizing cultures. In contrast, Fe(II) in reduced smectite can be readily oxidized by oxygen and was therefore used only in anaerobic, nitrate-reducing cultures. The biotite (Bancroft) was obtained from Ward Scientific, and the NAu-2 smectite from the Clay Minerals Society. The biotite was ground in a mortar and pestle to obtain 20–40 μm flakes, and added to medium from an autoclaved stock suspension. To make chemically reduced smectite, a mineral suspension in bicarbonate buffer (0.25 g/L NaHCO_3_ under 80:20 N_2_:CO_2_ atmosphere) was reacted with 10 mg sodium dithionite at 70°C for 20 min, washed 5 times with anoxic water, and sterilized by autoclaving.

#### In situ “i-chip” enrichments

Fe(III)-reducing and Fe(II)-oxidizing enrichment cultures were recovered from Hanford 300 groundwater through an *in situ* diffusion chamber incubation technique employing isolation chips (“i-chips”) developed by S.S. Epstein and colleagues. As discussed in detail in Bollmann et al. (2007) and Nichols et al. (2010), the diffusion-chamber-based approach is an alternative method of microbial isolation in which microorganisms form colonies in their natural environment, potentially allowing for isolation of organisms that could otherwise not be recovered by conventional culturing techniques. In this study, i-chips obtained from S.S. Epstein were used to enrich for either solid-phase Fe(III)-reducing or solid-phase Fe(II)-oxidizing organisms in Hanford 300 Area groundwater. This approach represents a novel adaptation of the i-chip approach, wherein electron donors and acceptors present *in situ* in the environment of interest are utilized as substrates for microbial growth. In our case, the mineral electron acceptors or donors and their microbial inocula were incubated *in situ* in order to obtain enrichments grown under conditions as close as possible to those present in the natural sediment/groundwater environment. To enrich for Fe(III)-reducers, either oxidized Ringold sediment or the oxidized (native) form of specimen NAu-2 smectite were used added to diffusion chambers as source of Fe(III) as an electron acceptor. To enrich for Fe(II)-oxidizers, either biotite or fine-grained (<20 μm) reduced Ringold sediment were added to diffusion chambers as source of solid-phase Fe(II) as an electron donor. The chambers allowed for a free exchange of solutes from Hanford groundwater via diffusion, while restricting the ingress or egress of microbial cells.

Groundwater was collected on 10/15/09, and on the following day diluted in agarized medium, mixed with corresponding mineral phase and loaded into the i-chip diffusion chambers [see Figure 1 in Nichols et al. (2010)]. Table 1 lists the composition of the i-chips, and Figure A1 shows where they were incubated *in situ*; only i-chips B1 and B3 were deployed above the redox boundary. To concentrate microorganisms for use in chip B3, groundwater was filtered through 0.2 μm filter and then the filter was washed in 1/10 of the initial volume of groundwater. On 10/21/09 the chambers were returned to Hanford 300 Area groundwater, suspended on PVC rods ca. 2–3 m below (for Fe(III)-reducers) or ca. 1 m above (for Fe(II)-oxidizers) the redox transition within the Ringold formation. Both oxygen and nitrate are present as electron acceptors above the redox transition, but are completely depleted below it (Lin et al., 2012a). The chambers were recovered on 03/01/2010, after ca. 5 months of *in situ* incubation.

**Table 1:** **Composition of i-chips used for *in situ* microbial cultivation**.

**Target microbial group**	**i-chip label**	**Agarized suspension loaded**
Fe(III) reducers	A1	Oxidized Ringold sediment + groundwater
	A2	Oxidized NAu-2 smectite + groundwater
Fe(II) oxidizers	B1	Reduced Ringold sediment + groundwater
	B3	Biotite + 10X concentrated groundwater

Individual colonies (960 total) were removed from the holes of the i-chips in an anaerobic chamber using sterile unfolded paper clips. Colonies were transferred into pressure tubes containing 2 mL of medium. For Fe(III) reducers, colonies were transferred to medium containing acetate (together in some cases with pyruvate and malate) and H_2_ as electron donors, and either (1) Fe(III) complexed with nitrilotriacetic acid [Fe(III)-NTA, prepared as described in Roden and Lovley (1993)], (2) oxidized NAu-2 smectite, or (3) a mixture of nitrate and fumarate as electron acceptors. For Fe(II) oxidizers, two types of media were utilized: (1) anaerobic medium with reduced NAu-2 smectite serving as the electron donor and 5 mM nitrate as the electron acceptor and (2) aerobic medium with Bancroft biotite serving as the electron donor. The NaHCO_3_-buffered medium described above was used for the anaerobic cultures, whereas the Pipes-buffered basal culture medium was utilized for all aerobic cultivations. Three mL of filtered air was added to each aerobic culture.

### Recovery of microbial isolates

Anaerobic enrichments were followed up with isolation procedures using a roll-tube method based on the original “Hungate technique” (Hungate, 1969). BBL agar (Becton Dickinson, Cockeysville, MD; 1.5%) was used as the solidifying agent. A 1-mL inoculum from 10-fold serial dilutions of an enrichment culture was added to 25 mL pressure tubes containing 7 mL of melted medium, and the tubes were rolled with a tube spinner (Bellco Glass, Inc.). For Fe(III)-reducing cultures, freshly-synthesized amorphous Fe(III) oxide (final concentration ca. 100 mmol L^−1^) was utilized as the electron acceptor and hydrogen (5 mL added to the head space) plus 5 mM acetate were utilized as the combined electron donor. For Fe(II) oxidizing cultures, Fe(II) complexed with nitrilotriacetic acid (Fe(II)-NTA) was utilized as the electron donor, with 5 mM nitrate as the electron acceptor. The Fe(II)-NTA stock solution was created by mixing equimolar amounts of FeCl_2_ and sodium NTA followed by filter sterilization. The Fe(II)-oxidizing cultures were set up with and without 2 mM acetate as a carbon source. After ca. 1 month of incubation, isolated colonies were transferred from roll-tubes to pressure tubes containing 2 mL of liquid medium of the same composition.

Two approaches were used to isolate aerobic Fe(II)-oxidizers. To isolate chemolithautotrophic organisms, enrichment cultures were serially diluted on a lithotrophic Pipes-buffered medium with aqueous Fe(II) as the sole energy source. An aliquot of FeCl_2_ (1.3 mmol L^−1^) and oxygen (1 mL of filtered air) were added every 2–3 days, and the cultures were incubated under static conditions, such that transfer of O_2_ into the liquid phase was controlled by diffusion. The highest positive dilutions, as indicated by significant cell growth, were serially diluted again, and this procedure was repeated three times.

To isolate mixotrophic microorganisms (i.e., lithotrophic microorganisms that can alternatively grow in heterotrophic medium), Fe(II)-oxidizing enrichment cultures were diluted to extinction on heterotrophic plates supplemented with 5 mM acetate and 0.05% yeast extract as the combined carbon and energy source. The resulting heterotrophic isolates could be either mixotrophic Fe(II)-oxidizers or heterotrophic contaminants. The numerically dominant colony types were tested for growth with FeCl_2_ as a Fe(II) source as described above for isolation of chemolithoautotrophic Fe(II) oxidizers. Cultures capable of growing to a density of at least 10^8^ cells mL^−1^ in either microaerophilic or anaerobic nitrate-reducing medium were selected for further study.

### Analytical techniques

Aqueous plus solid phase Fe(II) were quantified with the ferrozine assay after 1 h 0.5 M HCl extraction. This extraction recovers most (ca. 70%) of the Fe(II) content of reduced smectite (Jaisi et al., 2007), whereas only ca. 10% of the Fe(II) content in biotite is released (Shelobolina et al., 2012b). However, the HCl extraction procedure provides a convenient means to follow enzymatic oxidation of these mineral phases (Shelobolina et al., 2003, 2012b). In order to separate liquid and solid phases, aliquots of Fe(II)-oxidizing cultures were centrifuged in the anaerobic chamber prior to Fe(II) analysis. Centrifugation and removal of the supernatant was necessary to avoid potential chemical Fe(II) oxidation by nitrite under acidic conditions of HCl extraction (Sorensen and Thorling, 1991). Nitrate, nitrite, and acetate concentrations were measured using Dionex DX-100 ion chromatography (Dionex Corp., Sunnyvale, CA) with a Dionex AS4-SC IonPac column. Cells were counted with DAPI staining and epifluorescence microscopy (Hobbie et al., 1977).

16S rRNA gene sequences of isolated organisms were obtained using standard methodologies as previously described (Shelobolina et al., 2007). Genbank accession numbes for each of the isolates are given in Table 4. 16S rRNA genes were amplified using the GM3 and GM4 primer set (Muyzer et al., 1995). The 16S rRNA gene fragments were compared to the Genbank nucleotide database using BLASTN and BLASTX algorithms (Altschul et al., 1997). 16S rRNA gene sequences of the isolates were imported into ARB software package (Ludwig et al., 2004), which was merged with Greengenes database (November 2008 version), the clone library database of the Hanford Site subsurface sediment (Lin et al., 2012b), and the short-read sequences of Hanford formation groundwater bacteria (Lin et al., 2012c). The nearest reference strains and the previous Hanford sequences were identified and included in phylogenetic tree construction. A bootstrap-supported neighbor-joining tree was created based on evolutionary distances computed using the Kimura 2-parameter method in MEGA (Tamura et al., 2007).

### Growth experiments

A Fe(III)-reducing isolate was grown in NaHCO_3_-buffered medium containing ca. 6 mM acetate with the oxidized form of NAu-2 smectite [ca. 15 mmol Fe(III) L^−1^] as the electron acceptor. Aerobic Fe(II)-oxidizing isolates were grown in Pipes-buffered medium with pulsed additions of FeCl_2_ and oxygen as described above. Aerobic cultures with biotite [ca. 3 mmol L^−1^ of HCl-extractable Fe(II)] as the energy source were initiated with an inoculum from FeCl_2_/oxygen medium, and transferred several times in Pipes-buffered medium with monitoring of Fe(II) loss over time. Growth via nitrate-dependent oxidation of reduced smectite was conducted in NaHCO_3_-buffered medium containing ca. 5 mmol L^−1^ of HCl-extractable Fe(II) and 6 mM nitrate.

## Results and discussion

### Enrichment and isolation

**Figure A3:**
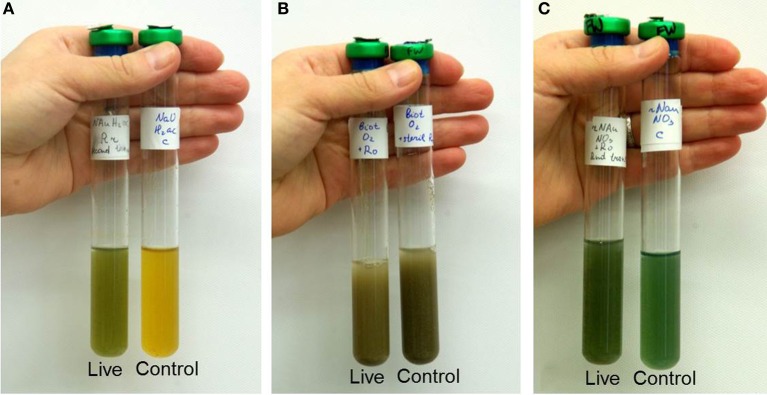
**Photos of (A) second transfer of NAu-2 smectite-reducing enrichment from reduced Ringold sediment (8.3 mmol Fe(II) L^−1^ in live vs. 0.3 mmol Fe(II) L^−1^ in control; **(B)** primary biotite-oxidizing enrichment from oxidized Ringold material (2.3 mmol Fe(II) L^−1^ in live vs. 10.5 mmol Fe(II) L^–1^ in control; **(C)** second transfer of reduced NAu-2-oxidizing enrichment from oxidized Ringold material (6.0 mmol Fe(II) L^−1^ in live vs. 12.7 mmol Fe(II) L^−1^ in control)**.

A large fraction (70–100%) of the 480 colonies transferred from i-chips targeting Fe(III) reducers resulted in the recovery of positive second-generation enrichments capable of utilizing either soluble Fe(III)-NTA, oxidized NAu-2 smectite, or a mixture of nitrate and fumarate as an electron acceptor (Table 2). Although none of the i-chip-derived Fe(III)-reducing enrichments were brought into pure culture, a Fe(III) phyllosilicate-reducing isolate designated strain R1 was recovered from conventional enrichments initiated with natural oxidized Ringold sediment (obtained from just above the redox transition zone) as the electron acceptor (see Table 4). The presence of active smectite reduction activity in enrichments (and the pure culture) was evidenced by a distinct color change in the mineral suspension (Figure A3A). The Fe(III)-reducing isolate is 98.9% similar in 16S rRNA gene sequence to *Geobacter bremensis* (Straub et al., 1998; Straub and Buchholz-Cleven, 2001), and is therefore referred to hereafter as *G. bremensis* strain R1. *G. bremensis* was originally isolated from freshwater ditch sediments in Germany (Straub et al., 1998; Straub and Buchholz-Cleven, 2001), and belongs to the “*Geobacter* subsurface clade 1” within the *Geobacteraceae* (Lovley et al., 2011).

**Table 2:** **Microbial recovery from i-chips A1 and A3 (see Figure A1) targeting Fe(III)-reducing microorganisms**.

**Medium**		**i-chip**	**Colonies transferred**	**Cultures recovered**	**% recovery^a^**
**Electron donor(s)**	**Electron acceptors(s)**				
Acetate + hydrogen	Fe(III)-NTA	A1	100	71	71
		A2	100	96	96
Acetate + hydrogen	Smectite	A1	100	97	97
		A2	100	100	100
Acetate + pyruvate + malate + hydrogen	Nitrate + fumarate	A1	30	29	97
		A2	50	39	78

a *Recovery was calculated as % of positive cultures on specific medium*.

The fractional recovery of lithotrophic Fe(II)-oxidizing enrichments from i-chip colony transfers (480 total) was much lower (7–10%) than in the case of Fe(III)-reducers, and was also much lower than that achieved when parallel i-chip colonies were transferred into heterotrophic medium with nitrate plus fumarate as electron acceptors (Table 3). Nevertheless, a variety of Fe(II)-oxidizing isolates were ultimately recovered from i-chip as well as conventional sediment enrichments, using both lithoautotrophic and mixotrophic isolation strategies (Table 4). The presence of aerobic and nitrate-reducing mineral oxidation activity was evidenced by a distinct color change in the biotite and reduced smectite suspensions, respectively (Figures A3B,C). Three strains of *Bradyrhizobium japonicum* (one isolated lithoautotrophically and two isolated mixotrophically) and one each strain of and *Cupriavidus necator*, *Dechloromonas agitata*, and *Nocardioides* sp. (all isolated on mixotrophic medium) were chosen for further study. *B. japonicum*, *C. necator*, and *D. agitata* have been previously identified as nitrate-reducing Fe(II)-oxidizers (Chaudhuri et al., 2001; Shelobolina et al., 2012a), whereas to our knowledge this is a first report of a Fe(II)-oxidizing *Nocardioides* species.

**Table 3:** **Microbial recovery from i-chips B1 and B3 (see Figure A1) targeting Fe(II)-oxidizing microorganisms**.

**Medium**		**i-chip**	**Colonies transferred**	**Cultures recovered**	**% recovery^a^**
**Electron donor(s)**	**Electron acceptors(s)**				
Chemically reduced NAu2 smectite	Nitrate	B1	100	7	7
		B3	100	10	10
Biotite	Oxygen	B1	100	3	3
		B3	100	9	9
Acetate + pyruvate + malate + hydrogen	Nitrate + fumarate	B1	20	12	60
		B3	20	10	50

a *Recovery was calculated as % of positive cultures on specific medium*.

**Table 4:** **Fe redox cycling microorganisms isolated from Hanford 300 sediments**.

**Strain designation (genbank accession number), Fe redox metabolism**	**Original source**	**Initial enrichment**	**Isolated on**	**No. of related isolates recovered**	**Identification (closest cultured bacterium, % identity)**	**Related 16S rRNA gene detected in sediment?**
*Geobacter bremensis* R1 (KF800712), Fe(III) reduction	Enrichment, oxidized Ringold sed + acetate/H_2_	Smectite + acetate/H_2_	HFO/acetate roll tubes	4	*Geobacter bremensis* TMJ1^T^, 98.9%	Yes^b^
					*Geobacter bemidjiensis*, 97.6%	
*Bradyrhizobium japonicum* 22 (KF800709), Fe(II) oxidation	i-chip, biotite	Biotite + O_2_	FeCl_2_/O_2_	10+	*Bradyrhzobium liaoningense* 2281^T^, 99.4%	Yes^a^
*Bradyrhizobium japonicum* is5 (KF800707), Fe(II) oxidation	i-chip, biotite	Biotite + O_2_	Heterotrophic plates		*Bradyrhizobium japonicum* USDA 6^T^, 99.4%	
*Bradyrhizobium japonicum* in8p8 (KF800708), Fe(II) oxidation	Enrichment, reduced Ringold sed + NO^−^_3_	Biotite + NO^−^_3_	Heterotrophic plates			
*Cupriavidus necator* A5-1 (KF800713), Fe(II) oxidation	i-chip, biotite	Biotite + O_2_	Heterotrophic plates	3	*Cupriavidus necator* ATCC 43291^T^, 98.6% “*Ralstonia eutropha*” H16, 98.4%	Yes^a^
*Dechloromonas agitata* is5 (KF800710), Fe(II) oxidation	Enrichment, biotite + NO^−^_3_	Fe(II)-NTA + NO^−^_3_	Fe(II)-NTA/acetate/NO^−^_3_ roll tubes	2	*Dechloromonas agitata* CKB^T^, 99.6%	No
*Nocardioides* sp. in31 (KF800711), Fe(II) oxidation	Enrichment, biotite + NO^−^_3_	Fe(II)-NTA + NO^−^_3_	Fe(II)-NTA/acetate/NO^−^_3_ Roll tubes	1	*Nocardioides pyridinolyticus* OS4^T^, 97.9%	Yes^a^

*The % values indicate the degree of similarity in 16S rRNA gene sequence*.

a *16S rRNA gene clone libraries (Lin et al., 2012b)*.

b *Quantitative PCR with Geobacter-specific primers (Lin et al., 2012a)*.

### Fe redox metabolism of the isolates

#### Fe(III) reducer

**Figure 1:**
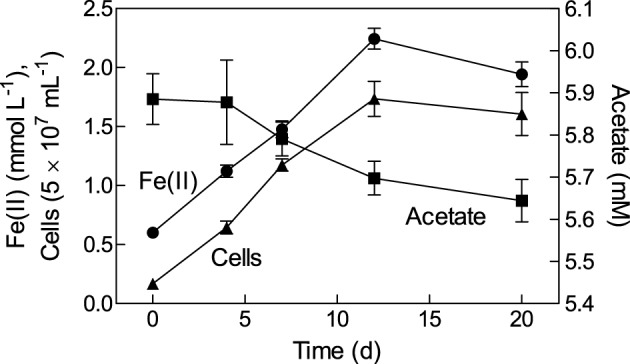
**Growth of *Geobacter bremensis* strain R1 with NAu-2 smectite as the electron acceptor and acetate as the electron donor**. Data represent mean ± SD of triplicate cultures.

A growth experiment with *Geobacter bremensis* strain R1 using oxidized NAu-2 smectite as the electron acceptor and acetate as the electron donor showed direct coupling of cell growth to Fe(II) production and acetate consumption (Figure 1). The quantity of acetate consumed was approximately equal to the value of 0.2 mM expected for reduction of ca. 1.6 mmol L^−1^ of Fe(III). Approximately 14% of the total Fe(III) content of the smectite was reduced, comparable to values obtained in other microbial Fe(III) phyllosilicate reduction studies (Kostka et al., 1999; Shelobolina et al., 2003; Jaisi et al., 2005; Komlos et al., 2008; Mohanty et al., 2008). The isolate can also grow with amorphous Fe(III) oxide or fumarate as the electron acceptor (data not shown).

#### Fe(II) oxidizers

**Figure 2:**
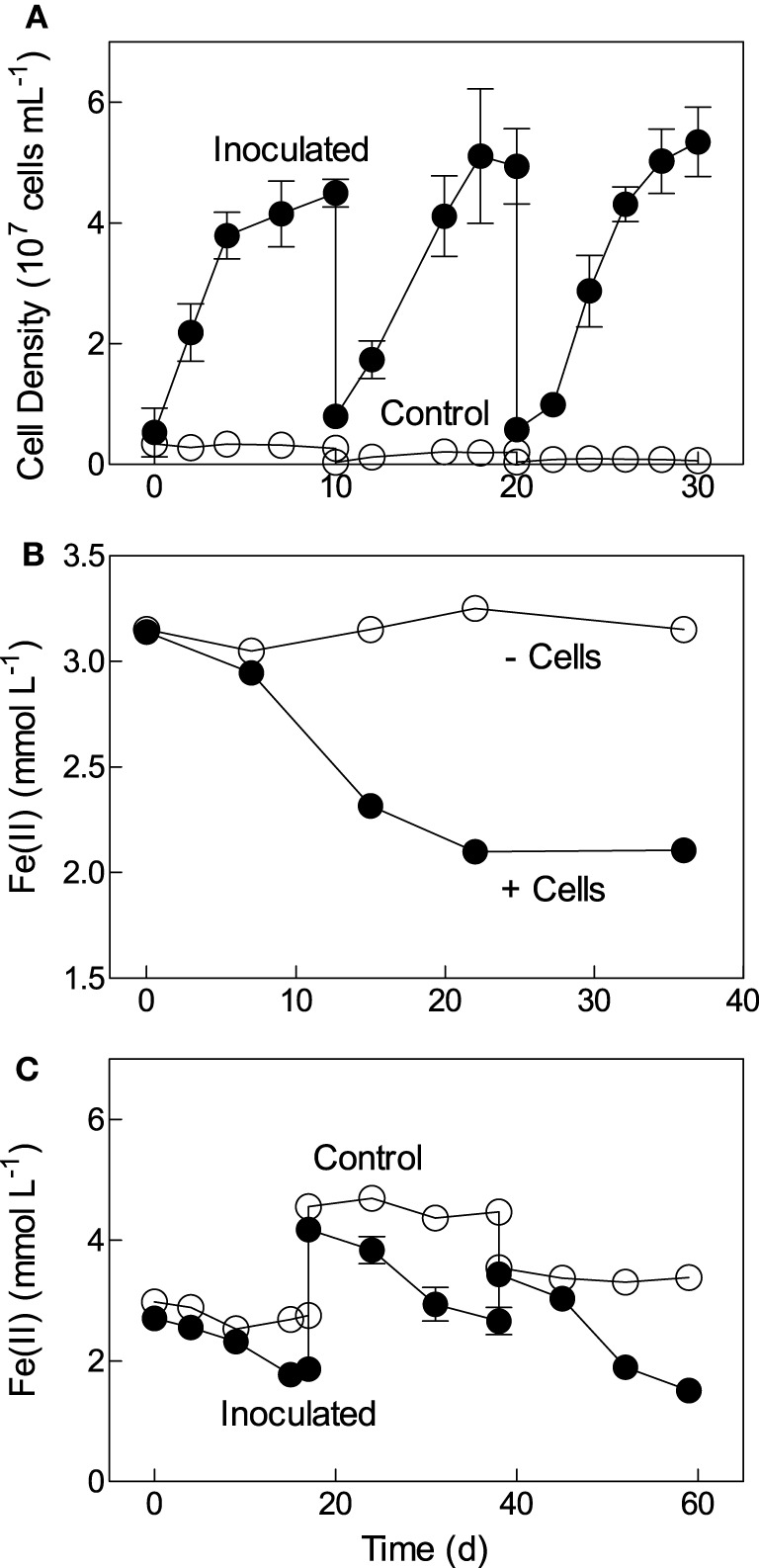
**Fe(II) oxidation by *Bradyrizobium* sp. strain 22: (A) Repeated growth in aerobic FeCl2 medium; (B) aerobic oxidation of biotite (inoculum grown previously several times in aerobic biotite medium); (C) repeated growth in reduced NAu-2/nitrate medium**. Data in panel A show the mean ± SD of five replicate cultures; data in panel **(B)** represent the results from a single culture that had been transferred several times in identical medium before conducting this experiment; data in panel **(C)** show the mean ± SD of triplicate cultures.

**Figure 3:**
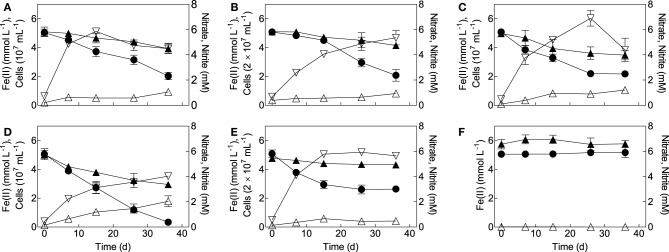
**Growth of mixotrophic Fe(II)-oxidizing isolates on reduced NAu-2 smectite with nitrate as the electron acceptor. (A)** *Bradyrhizobium* sp. strain in8p8; **(B)** *Bradyrhizobium* sp. strain bis5; **(C)** *Cupriavidu necator* strain A5; **(D)** *Dechloromonas agitata* strain dis5; **(E)** *Nocardioides* sp. strain in31; **(F)** sterile control. Data represent the mean ± SD of triplicate cultures. Symbols: •, Fe(II); ▴, nitrate; ▵, nitrite; ▿, cells.

*Bradyrhizobium japonicum* strain 22 was derived from aerobic biotite enrichment cultures initiated with reduced Ringold sediment, and isolated under chemolithoautotrophic conditions via dilution to extinction in FeCl_2_/O_2_ medium. Subsequent studies confirmed that strain 22 was capable of repeated chemolithoautotrophic growth with soluble Fe(II) as the sole electron donor and oxygen as the electron acceptor (Figure 2A). The cell yield in these experiments was approximately 5 × 10^7^ cells per μmol Fe(II) oxidized, assuming that most of the Fe(II) oxidation took place biologically, which is typically the case in non-mixed, diffusion-controlled Fe(II) oxidation experiments such as those employed here (Sobolev and Roden, 2001; Roden et al., 2004). This cell yield is comparable (within a factor of 2–3) to that observed for other neutral-pH chemolithoautotrophic Fe(II) oxidizing bacteria (Neubauer et al., 2002; Sobolev and Roden, 2004). Strain 22 could also aerobically oxidize structural Fe(II) in biotite (Figure 2B), and repeatedly oxidized structural Fe(II) in reduced NAu-2 smectite with nitrate as the electron acceptor (Figure 2C). The extent of biotite oxidation (ca. 5% of total mineral Fe(II) content) was similar to that observed during growth of the chemolithoautotrophic Fe(II)-oxidizing, nitrate-reducing “Straub culture” with biotite as the energy source (Shelobolina et al., 2012b), and the extent of reduced smectite oxidation (ca. 40%) was comparable to that observed for the other Fe(II)-oxidizing isolates described here (see Figure 3).

**Figure A4:**
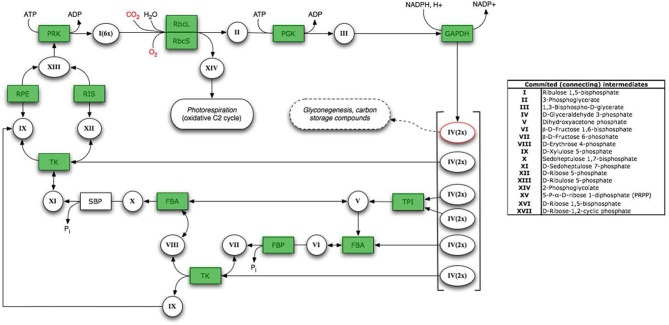
**Calvin-Benson subsystem in *B. japonicum* strain 22, generated by SEED Viewer version 2.0 within the RAST annotation software**. Enzymes indicated in green boxes are present in the genome. Abbreviations: PRK, Phosphoribulokinase; RbcL, Ribulose bisphosphate carboxylase large chain; RbcS, Ribulose bisphosphate carboxylase small chain; PGK, Phosphoglycerate kinase; GAPDH, NAD-dependent glyceraldehyde-3-phosphate dehydrogenase; TPI, Triosephosphate isomerase; FBA, Fructose-bisphosphate aldolase; FBP, Fructose-1,6 bisphosphatase; TK, Transketolase; RPE, Ribulose-phosphate 3-epimerase; RIS, Ribose 5-phosphate isomerase.

Strains of *B. japonicum* are known to be capable of autotrophic growth with H_2_ as the electron donor (Neal et al., 1983; Franck et al., 2008). In addition, *B. japonicum* strain USDA110 is capable of chemolithotrophic growth with thiosulfate as the sole electron donor (Masuda et al., 2010). Although Fe(II)-phyllosilicate oxidizing strains of *Bradyrhizobium* were recently isolated from a clay-rich subsoil in Wisconsin (Shelobolina et al., 2012a), the NAu-2 smectite employed in that as well as the present study was not completely free of associated organics. Thus, our studies with FeCl_2_/O_2_ medium represent the first demonstration of the ability of *B. japonicum* to grow via Fe(II) oxidation under fully chemolithoautotrophic conditions. The inferred capacity for CO_2_ fixation was confirmed through preliminary whole genome sequencing of strain 22 genomic DNA at the University of Wisconsin Biotechnology Center. The Illumina sequence was assembled de novo using the CLC Genomics Workbench, and annotated through RAST (Version 4.0). The annotated genome revealed the presence of the entire Calvin-Benson CO_2_ fixation subsystem (see Figure A4).

All of the organisms isolated as mixotrophs (*B. japonicum* strains is5 and in8p8, *D. agitata* strain is5, *C. necator* strain A5-1, and *Nocardioides* sp. strain in31) grew in medium with chemically reduced NAu-2 smectite as the electron donor and nitrate as the electron acceptor (Figure 3). Each strain was grown twice in the reduced smectite/nitrate medium prior to conducting the growth experiments shown in the Figure 3. Growth generally ceased after 15–36 days, when there was still 0.5 M HCl-extractable Fe(II) present in the medium. The incomplete oxidation of structural Fe(II) in smectite has been observed previously in abiotic (Shen and Stucki, 1994; Yang et al., 2012) and biotic oxidation studies (Shelobolina et al., 2012a); a possible explanation for this phenomenon is that collapse of smectite layers during Fe(III) reduction makes a portion of the structural Fe(II) inaccessible to subsequent abiotic or enzymatic attack (Stucki, 2011). There was modest accumulation of nitrite (ca. 0.5–1.5 mM) during Fe(II) oxidation, and thus abiotic reaction of nitrite with reduced NAu-2 smectite could have contributed to the observed Fe(II) oxidation activity. However, recent studies of NAu-2 oxidation by organisms isolated from clay-rich subsoils showed that the kinetics of this abiotic reaction are such that enzymatic oxidation is the predominant mechanism for nitrate-driven smectite oxidation (Shelobolina et al., 2012a). This conclusion is supported by the cell yields in these experiments, which varied from 1.5–5 × 10^7^ cells per upmuol Fe(II) oxidized, well within the range observed for growth of *B. japonicum* strain 22 and other aerobic Fe(II) oxidizers in FeCl_2_/O_2_ medium (see above), as well as nitrate-dependent growth of the chemolithoautrophic Fe(II)-oxidizing “Straub culture” with aqueous or solid-phase Fe(II) as the sole energy source (Blöthe and Roden, 2009; Shelobolina et al., 2012b).

### Relevance of the isolates to in situ phyllosilicate Fe redox metabolism

**Figure 4:**
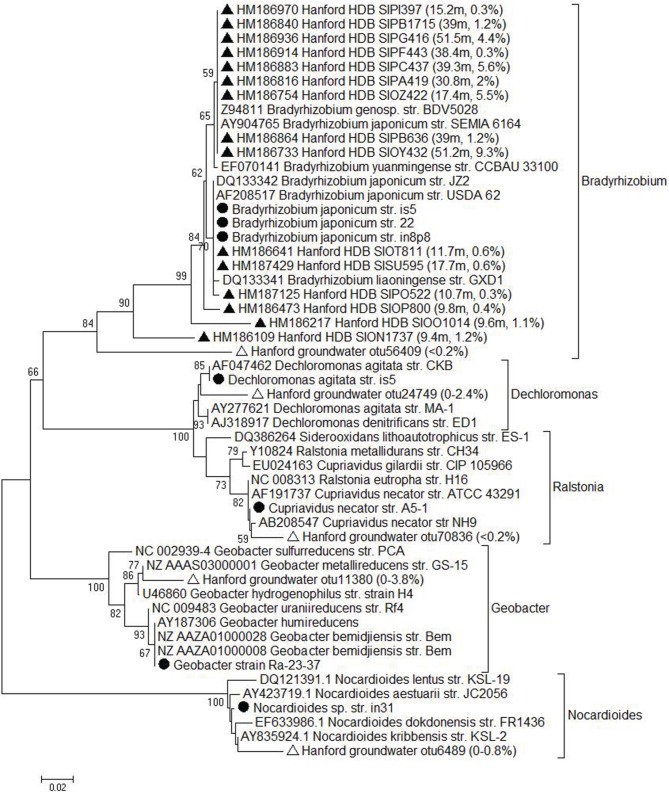
**Neighbor-joining tree of16S rRNA gene sequences for the isolates (circles) with the presence of the nearest reference strains and the Hanford sediment (solid triangles) and groundwater (open triangles) clones**. Bootstrap values less than 50% were not shown. Numbers in parenthesis indicate the depth of the Hanford sediment sample in meter and the percentage of this clone/sequence in each clone library at the specific depth or the range of their relative abundance in groundwater pyrosequencing library.

16S rRNA gene sequences of the Fe(III)-reducing and Fe(II)-oxidizing isolates were compared to sequences contained in the Greengenes (Desantis et al., 2006) database, which was augmented with the Hanford 300 Area subsurface sediment full-length 16S rRNA gene clone library database from Lin et al. (2012b), as well as a 16S rRNA gene 454 pyrosequence amplicon database for Hanford 300 Area of groundwater bacteria (Lin et al., 2012c). A bootstrap-supported neighbor-joining tree (Figure 4) was created based on evolutionary distances computed using the Kimura 2-parameter method in MEGA (Tamura et al., 2007). All of the isolates were related, at least to the genus level, to taxa identified in conventional and pyrosequencing libraries of 16S rRNA genes from the Hanford 300 Area subsurface. Thus, our enrichment and isolation studies successfully recovered organisms related to those previously identified by culture-independent approaches.

The recovery of a Fe(III) phyllosilicate-reducing *Geobacter* isolate from Ringold formation sediments was not unexpected given that Fe(III) phyllosilicates contribute a significant portion of Fe(III) in Ringold oxidized sediment (Peretyazhko et al., 2012), and that multiple species of *Geobacteraceae* are known to reduce structural Fe(III) in phyllosilicates (Shelobolina et al., 2007). Organisms from the *Geobacteraceae* were shown to be present in relatively high abundance (as indicated by qPCR analysis of 16S rRNA genes) in the vicinity of the redox transition in the upper Ringold formation (Lin et al., 2012a). The facile recovery of active Fe(III) phyllosilicate-reducing enrichments from i-chip colony transfers (Table 2) is likewise consistent with the presence of a Fe(III)-reducing community in the vicinity of the redox transition in Ringold sediments. In contrast to these findings, recent studies of the potential for Fe(III) reduction in Ringold formation sediments (from both above and below the redox transition) with and without added organic carbon (0.9 mM acetate, 0.6 mM lactate, and 0.3 mM glucose) yielded negative results (Lee et al., 2012). There is no obvious reason for this discrepancy, as our initial enrichment culturing showed substantial reduction (14–21%) of oxidized Ringold sediment by native Fe(III)-reducing populations. In addition, studies of the potential for phyllosilicate Fe redox cycling in Ringold sediment employing a pure culture of *G. sulfurreducens* have verified that Fe(III) phases in oxidized Ringold sediment are available for microbial reduction (Shelobolina et al., unpublished data), and recent microcosm experiments have demonstrated the potential for reduction of Fe(III) phases in Ringold sediment from just below the redox transition (Percak-Dennett and Roden, unpublished data). It seems possible that heterogeneities in sediment subsamples used in different experiments could account for the lack of Fe(III) reduction in Ringold sediments reported by Lee et al. (2012).

We recovered a suite of lithotrophic organisms capable of oxidizing structural Fe(II) in smectite with nitrate (or, in some cases, biotite with O_2_) as the electron acceptor from Hanford 300 Area sediments and groundwater, all of which have been detected in previous molecular surveys. In particular, *Bradyrhizobium*-related taxa constituted a significant fraction (up to 5%) of 16S rRNA gene sequences in clone libraries from sediments above the redox transition (see Figure 4). Does this imply that such organisms are playing an active role in Fe silicate mineral redox cycling in Hanford sediments? Although the isolates reported here were not screened for their ability to oxidize native reduced Fe(II) phases present in Ringold formation sediments, experiments with the chemolithoautotrophic Fe(II)-oxidizing, nitrate-reducing “Straub culture” [which is capable of oxidizing structural Fe(II) in both biotite and smectite; Shelobolina et al. (2012b); Xiong (2013)] indicate that such phases are in fact susceptible to partial enzymatic oxidation. Thus, it seems feasible that Fe(II)-oxidizing lithotrophs could gain energy from oxidation of the large quantities of structural Fe(II) present in reduced Ringold sediments. Recent sediment microcosm experiments with reduced Ringold sediments have demonstrated the potential for partial biologically-mediated oxidation of solid-phase Fe(II) with nitrate as the electron acceptor (Percak-Dennett and Roden, unpublished data).

## Conclusion

A culturing campaign successfully recovered Fe-phyllosilicate redox cycling organisms from sediments and groundwater in the vicinity of a distinct redox transition in the Hanford 300 Area subsurface. The recovered organisms are phylogenetically related to organisms detected in 16S rRNA gene libraries for Hanford 300 Area sediments. Hence, the isolates represent appropriate targets for further physiological and genomic studies of Fe-phyllosilicate redox metabolism relevant to the Hanford subsurface. To this end, each of the Fe(III)-reducing and Fe(II)-oxidizing isolates described above are currently undergoing whole genome sequencing through the U.S. Department of Energy's Joint Genome Institute (JGI) Microbial Isolates sequencing program. The results of this project will expand significantly our knowledge of the diversity of lithotrophic Fe(II) oxidation metabolism. Of particular interest is the mechanism(s) by which the Fe(II)-oxidizing taxa utilize Fe(II) in insoluble Fe-silicate minerals such as smectite and biotite. These minerals are virtually insoluble at neutral pH, which means that the organisms must possess specific machinery to extract electrons from the mineral surface. The emerging picture of how neutral pH Fe(II) oxidizers and Fe(III) reducers may utilize analogous strategies to carry-out extracellular electron transfer (Hartshorne et al., 2009; Bird et al., 2011; Liu et al., 2012; Roden, 2012) will be informed and expanded by the sequencing project. Development of genome-enabled techniques to detect the presence and expression of genes associated with solid-phase Fe(II) oxidation will eventually provide direct insight into the influence of enzymatic Fe(II) oxidation on biogeochemical processes in Hanford 300 Area and other subsurface environments.

### Conflict of interest statement

The authors declare that the research was conducted in the absence of any commercial or financial relationships that could be construed as a potential conflict of interest.
